# The Impact of Artificial and Natural Additives in Meat Products on Neurocognitive Food Perception: A Narrative Review

**DOI:** 10.3390/foods13233908

**Published:** 2024-12-03

**Authors:** Marius-Mihai Ciobanu, Elena-Iuliana Flocea, Paul-Corneliu Boișteanu

**Affiliations:** 1 Department of Food Technology, ‘Ion Ionescu de la Brad’ Iasi University of Life Sciences, 700490 Iasi, Romania; marius.ciobanu@iuls.ro; 2 Faculty of Food and Animal Sciences, “Ion Ionescu de la Brad” University of Life Sciences, 8 Mihail Sadoveanu Alley, 700489 Iasi, Romania; paul.boisteanu@iuls.ro

**Keywords:** meat products, artificial additives, natural additives, organoleptic properties, food perception

## Abstract

The holistic sensory experience creates a unified perception that influences consumer memory. Consumer interest in clean label products underlines an accelerated trend towards products without artificial additives. From a sensory point of view, food appeal is significantly influenced by how additives actively participate in the organoleptic properties of the final product. This research aims to shed light on the impact of artificial and natural additives in meat products on neurocognitive food perception, which is essential for understanding how they influence the consumer’s final decision and direct food trends. Different neural mechanisms involved in multimodal sensory integration confirm differences in perception of meat products containing artificial and natural additives. Analysis confirms that neurocognitive perception integrates organoleptic sensations to form a complete sensory experience. The encephalon simultaneously processes multimodally integrated stimuli from organoleptic properties, reaching the orbitofrontal cortex and other regions involved in the neuroprocessing of the final product. The reformulation and development of meat products need a detailed analysis of the impact of additives on sensory properties contributing to the shaping of consumption trends.

## 1. Introduction

**Figure 1:**
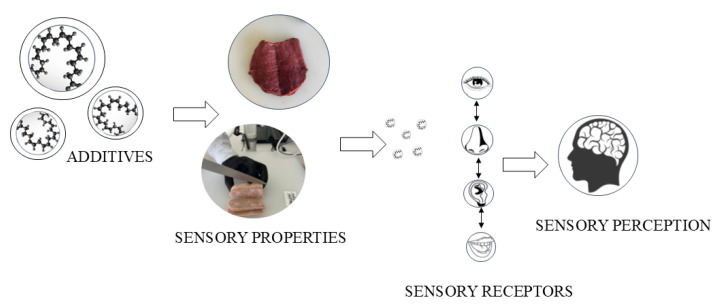
Multimodal sensory integration of sensory properties resulting from the use of additives in meat products. Processing content according to Chen J. [9].

Fresh and processed meat offer numerous nutritional health benefits, they are high in protein, balanced in amino acids, and rich in several minerals and vitamins, which play an essential role in metabolism and can be more easily assimilated from meat than from other foods [1]. Meat and meat products offer a unique type of food satisfaction in modern society. Certain products require additives to ensure efficient industrial processing. The additives actively participate in preserving meat products and improving food safety, but they also enrich sensory properties. Utilization of additives such as butylated butylated hydroxyanisole (BHA), butylated hydroxytoluene (BHT), propyl gallate (PG), and tertiary butylated hydroquinone (TBHQ) have long been used to inhibit harmful oxidation-induced changes in meat that cause sensory degradation, but are under increasing scrutiny due to potential genotoxic effects on human health [2]. Consumers associate artificial additives with harmful health effects; however, they are monitored constantly by The European Food Safety Authority (EFSA) in the EU and the FDA (Food and Drug Administration) in the USA. In the EU, for meat products, the list is long, including antimicrobials, antioxidants, and texturizers as the most commonly used, but also other additives (such as colourants, stabilizers, and acidity regulators). All additives used in meat processing are considered safe within the limits set by food safety authorities [3,4]. The current direction is towards natural antioxidants derived from various plant materials rich in polyphenols. The authors in [5] emphasized the antioxidant properties of natural extracts that are proposed as potential replacements for synthetic additives such as nitrites in meat products, further highlighting the impact of additives on sensory properties. Those in [6] supported that the application of natural additives in meat products can maintain similar sensory characteristics as those obtained by artificial additives, but more studies are needed to maximize their sensory productivity in the final product. Food preference and perceptual differences for meat products containing natural or synthetic additives may be distributed over different neural mechanisms responsible for multimodal sensory integration [7]. Fluctuations in neurophysiological states in the sensory neocortex play an important role in perceptual variability, influencing the processing of sensory stimuli and contributing to perceptual differences [8]. Figure 1 illustrates the process of multimodal sensory integration of sensory experience of the consumer. The Figure 1 summarizes how additives alter the sensory experience of the consumer. It demonstrates how chemical interventions influence the physical and organoleptic properties of the final product, which in turn activate complex sensory processes. Figure 1 starts with a symbolic representation of the chemical structure of food additive. Additives are added to meat products to modify or enhance the sensory characteristics of meat products intended for consumption. This schematic representation between the molecules released by meat and human sensory receptors illustrates how chemical signals are transmitted and interpreted by the sensory system. The brain image on the right suggests the integration of sensory signals. The brain processes and combines information from different receptors to generate an overall perception of the product.

Human ascending sensory systems are involved in food perception. In multimodal sensory integration, the visual system participates through distal recognition and food selection. Striate and extrastriate areas of the ventral system provide information on external appearance. As one progresses from the striate primary visual cortex to the visual inferotemporal areas of the temporooccipital cortex and temporal-anterior cortex, the size of the visual receptive fields and the optimal stimuli become increasingly complex. The visual projections of the inferotemporal cortex move to the amygdala, orbitofrontal cortex, and adjacent perirhinal cortex [10]. Studies analysing food perception generate distinct neuronal activations in the medial prefrontal cortex (MPFC). The medial prefrontal cortex is associated with complex cognitive processes, including preference formation and decision-making. By associating food with health benefits and a positive impact on sustainability, the brain activates neural pathways related to moral evaluation and reward. This phenomenon indicates that organic food perceptions are nuanced and highlights the complexity of brain processes involved in food perception and decision-making, going beyond simply recognizing taste or food appearance [11]. Perception of meat foods with natural and artificial additives involves distinct neural pathways and mechanisms. The brain simultaneously processes stimuli from the organoleptic properties, reaching the orbitofrontal cortex and other regions involved in the neuroprocessing of the flavour of the final product. Research suggests that brain activation during food consumption is characterized by the convergence of olfactory and gustatory sensations, leading to a holistic flavour experience. Depending on the type of additives, different activations in the brain may reflect their influence on how the meat product is perceived. Natural additives are associated with foods perceived as healthy, they may activate reward circuits in the brain more intensely than artificial additives that are associated with health problems. Artificial additives may induce lower or potentially ambiguous activations [12].

When the consumer does not consult the product label, artificial additives show a higher sensory efficacy, favouring acceptance and increased interest in the product compared to natural additives, which have relatively lower sensory efficacy. The sensitivity of the brain to visual cues of spoiled or rotten foods indicates an automatic mechanism for detecting inedibles, triggering enhanced attention capture and early alarm responses [13]. The brain processes and differentiates meat foods with natural and synthetic additives through early visual discrimination and neural responses. The neural responses differ for foods with natural additives compared to those processed with artificial additives, reflecting instantaneous assessments of the perceived value and suitability of the food essential for consumption decisions. Research shows that the brain can distinguish between food and non-food products within milliseconds of stimulus onset, encoding information about the naturalness of the food, the level of processing, and the caloric content. Regardless of the attention or cognitive task imposed, this rapid processing occurs as a function of an automatized mechanism, whereby the encephalon rapidly evaluates possible food sources to determine food safety and consumption decisions [14].

Under the influence of insulin, which plays a modulatory role by amplifying or attenuating, cortical activity responds differently to food stimuli. Images of food with sensory-significant properties generate more intense neural activity compared to control images. Insulin contributes to sensory discrimination of food by adapting neural responses to food type and quality [15]. The brain’s reaction to meat products with artificial additives can be multi-sided. Synthetic food colourings, commonly used to enhance the attractiveness of processed foods, have been linked to an increase in behavioural problems in children, such as attention deficit disorder (ADD) and attention-deficit/hyperactivity disorder (ADHD) [16]. Additives, such as sodium nitrite and monosodium glutamate (MSG), used in meat processing, have been implicated in various pathological changes by damaging brain cells and may also affect male fertility [17]. These additives can challenge the immune system, leading to inflammatory responses and may cause neurobehavioral disorders. Therefore, the brain response to artificial meat additives may involve immune reactions, inflammatory cascades and neurological implications, highlighting the importance of understanding and monitoring the impact of these additives on brain perception and health [18]. The inclusion of meat products with artificial additives in the diets of laboratory animals affected by hemorrhagic strokes indicates a potential negative impact on brain tissue. The study shows that these additives may help to stabilize certain destructive changes in brain tissue, particularly in neurons through the proliferation of glial cells in response to injury. Gliosis indicates an attempt by the tissue to adapt and respond to harmful changes caused by artificial additives. This mechanism provides a possible detrimental impact of artificial additives on neuronal function and integrity under conditions of brain vulnerability [19]. On the other hand, studies suggest that the incorporation of natural extracts into meat products may help to reduce carcinogenic compounds by decreasing the formation of N-nitroso compounds [20]. Understanding the neural mechanisms involved in perception and preference may help to elucidate how individuals make food choices based on sensory cues and modification of nutritional value, as demonstrated in studies of food choices and neural activity during decision-making tasks [21].

This review explains consumer responses by exploring the impact of artificial and natural additives in meat products on neurocognitive perception. The integration of food neurocognitive perception provides a complex and interdisciplinary framework for the development and market success of reformulated meat products. The way natural and artificial additives influence neurocognitive mechanisms, and the perception of organoleptic properties of products explains consumption decisions. Analysing the potential of natural additives to replace artificial compounds is a key focus for food industry specialists without compromising the acceptability of reformulated products. Studying the link between food neuroperception and additives in meat products focuses on how added compounds guide consumption trends. This interdisciplinary field fosters innovation in food science.

## 2. Neurocognitive Mechanisms Involved in Food Perception

Food flavour molecules have the potential to influence people’s emotions via olfactory pathways. Current food flavour research mainly focuses on the physicochemical properties and formation mechanisms of flavour components, neglecting the effects of flavour molecules in emotional regulation. The assessment of liking, an essential dimension of emotion, lacks objective assessment methods. In one study [22], sensory evaluations of sensory acceptabilities for 12 aroma compounds were collected from 45 subjects, and their correlation with brain activity responses in the left frontal-temporal lobe (LFT) and right frontal-temporal lobe (RFT) were analysed. The results revealed a close relationship between brain activity in the left frontal-temporal lobe (LFT) and the perception of aroma liking. The authors confirm that they observed a substantial correlation between the α, β and γ frequency bands in the left frontal-temporal (LFT) and subjective pleasure scores. These findings demonstrate that the left frontal-temporal (LFT) plays a critical role in the evaluation of the pleasantness of flavour molecules and that changes in the strength of the α, β, and γ bands serve as important indicators of assessment [22]. Taste processing in primates involves several brain structures. The nucleus of the solitary tract and the primary gustatory cortex provide the direct perception of the characteristics of food by identifying and intensifying taste inputs (sweet, salty, sour, bitter, umami, etc). The orbitofrontal cortex is involved in taste evaluation, influenced by physiological states of the organism, such as hunger. The neuronal structures in this area respond to taste at the sensation of hunger and manifest sensory-specific satiety. In the orbitofrontal cortex, there is a convergence of olfactory and gustatory input, forming a complex perception of food. This region can integrate information about the texture and appearance of food. The orbitofrontal cortex allows rapid associations between taste and other sensory stimuli. This facilitates food recognition based on previous experience, having an important role in determining food learning preferences. The orbitofrontal cortex has a complex role in integrating and processing information about the organoleptic properties of the product, influencing consumer preferences and behaviour [23]. Neurophysiologic recordings and functional neuroimaging show the primary gustatory cortex in the rostral insula and adjacent frontal operculum provide separate and combined representations of taste, temperature, and texture of food. Neurons respond differently to combinations, providing a rich representation of the sensory properties of food. The representation of taste and other food-related stimuli is found mainly in the medial orbitofrontal cortex. Consistently, the activation of parts of the human orbitofrontal cortex correlates with subjective ratings of the pleasantness of food taste and odour. Food intake is mediated by medial orbitofrontal cortex activation through multimodal integration of the sensory properties of products. A neuronal representation of taste is also found in the pregenual cingulate cortex, which receives information from the orbitofrontal cortex, and, in humans, many pleasant stimuli activate the pregenual cingulate cortex, indicating that this is an area important in motivation and emotion [24]. Optogenetic activation of hypothalamic agouti-related hypothalamic peptide provides information on how internal states such as hunger influence odour attractiveness. Neuronal projections to the paraventricular thalamus contribute to sensory integration and regulation of olfactory stimuli. Neuropeptide Y plays an essential role in the selective activation of olfactory subcircuits and influences behaviour depending on the physiological state. The release of neuropeptide Y in the thalamus highlights how internal states can modulate behavioural preferences related to the olfactory sense [25].

**Figure 2:**
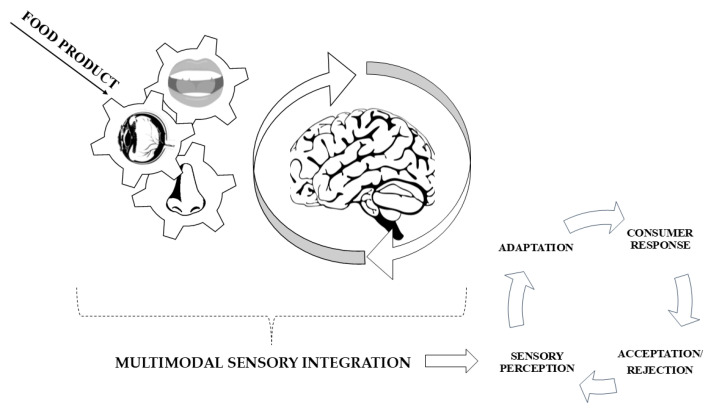
The general approach to multimodal sensory integration and consumer response. Processing content according to [24,25,26].

According to [26], differentiated perception of food odours activates medial prefrontal and lateral orbitofrontal areas. Medial prefrontal area activation to food odours and scores on the externalness subscale of the Dutch Eating Behaviour Questionnaire subscale under satiety conditions are positively correlated. In individuals who are sensitive to external food cues (organoleptic properties), the medial prefrontal area plays a role in the inappropriate evaluation of food stimuli even when hunger is reduced. Consumers influenced by external stimuli may continue to respond to sensory properties in a way that predisposes them to food consumption even in the absence of a physiological need [24,25,26]. Figure 2 illustrates a general approach to multimodal sensory integration and consumer response. The food product is the initial stimulus, it interacts with several sensory organs (eyes, nose, mouth) of the consumer. The information collected by the senses is integrated at the level of the brain. The brain combines the signals to form a unified perception of the food product. Sensory processing determines consumer responses by adapting the information. The consumer adjusts their perception over time, based on previous experiences. The response may be positive, with the consumer accepting the product for consumption, or it may be perceived negatively, rejecting consumption of the final product.

## 3. The Impact of Artificial Additives

Meat and processed meat products are valuable foods for consumers due to their sensory properties and the presence of essential nutritional components such as proteins with high biological value, minerals (Fe, Zn, Se), and some vitamins (mainly B6 and B12) [27]. Additives are intended to enrich the sensory properties of meat products, making them more attractive to consumers. Artificial additives in meat products play an essential role in preservation, colour, and flavour and inhibit bacterial spoilage [28]. Food additives can bring great sensory pleasure and marketing comfort to humans, but they may also cause potential risks to human health [29] through the production of methemoglobin and the potential carcinogenic effects of N-nitrosocompounds [30]. Phosphate additives are widely applied in food processing companies to improve product quality in a time-efficient manner [31]. Common additives in processed meat include nitrites, phosphates, and monosodium glutamate, which improve sensory qualities and shelf life and ensure production in an optimal time [32]. Calcium propionate, sodium nitrate, sodium nitrate, sodium benzoate, sodium nitrite, sulphites (sulphur dioxide, sodium bisulphite, potassium hydrogen sulphate, etc.) and disodium are examples of common chemical preservatives with a high teratogenic effect [33]. Ref. [34] confirmed the adverse effects of sodium nitrate by a dose-dependent decrease in sperm motility, serum testosterone concentration, body weight and organ weights and an increase in abnormal sperm morphology in *NaNO_3_-treated* groups. In addition, histologic analysis confirmed that *NO_3_* induced toxicity. Decreased seminiferous tubules and loss of spermatids in the testes, shrinkage of pancreatic acinar cells, sinusoidal congestion and necrosis of the liver, atrophy of glomeruli and congestion of the renal tubules of the kidney were the histologic changes observed.

One of the compounds known as a preservative with a high safety profile is sodium benzoate. While some studies show that it can be used to treat conditions such as depression, pain, schizophrenia, autism spectrum disorders, and neurodegenerative diseases, others report its harmfulness. For example, it has been found to cause mutagenic effects, generate oxidative stress, disrupt hormones, and reduce fertility [35]. Ref. [36] examined the effects of sodium benzoate, a common preservative, on sweet and sour taste perception in healthy adults. They used an alternative forced-choice and time-intensity method to measure the intensity and duration of sweet and sour sensations elicited by sucrose and citric acid solutions with or without sodium benzoate. They found that sodium benzoate significantly reduced the intensity and duration of both sweet and sour tastes, suggesting that it interferes with the activation of taste receptors and/or signal transduction pathways. They recommended consumers should be aware of the potential sensory effects of sodium benzoate and that food manufacturers should consider alternative preservatives or lower concentrations of sodium benzoate [36,37,38]. Due to the large number of reports on the harmfulness of food additives, more and more consumers are following the so-called “clean label” trend, i.e., preferring and choosing the least processed foods. Monosodium glutamate can be used to reduce sodium intake without compromising taste [37]. For sodium reduction, the use of salt substitutes, such as potassium chloride (*KCl*), is a classic alternative. However, *KCl* imparts bitter and metallic tastes to the product, which can be reduced by the use of flavour enhancers that mask these undesired tastes [37].

Table 1 presents a selection of natural substitutes that can replace most of the synthetic additives used in the meat industry, offering healthier and consumer-acceptable alternatives.

## 4. The Impact of Natural Additives

A major obstacle to the use of natural plant extracts in food has been the imposition of undesirable flavours and odours. However, technological advances have enabled food ingredient manufacturers to produce extracts with sensory characteristics that do not interfere while maintaining antioxidant properties. These technologies include solvent extraction, hydrodistillation, spray drying, and supercritical fluid extraction [41,42,43]. Antioxidants are used to minimize lipid oxidation. Antioxidants can act as metal chelators and free radical scavengers or oxygen scavengers, which can slow down the rate of lipid oxidation. Lipid oxidation can have negative effects on the quality of meat and meat products, causing changes in sensory attributes such as colour, texture, odour, and flavour as well as nutritional quality. Several synthetic antioxidants have been successfully used to prevent lipid oxidation in the meat industry, but consumers are concerned about the health risks of consuming certain synthetic antioxidants. Therefore, there is increased interest in natural antioxidants. Currently, compounds obtained from natural sources such as cereals, oilseeds, spices, fruits, and vegetables have been studied to reduce lipid oxidation [51,52]. Plants are rich in bioactive compounds (BACs), mainly polyphenols, which are valuable choices to replace synthetic antioxidants in meat products. These natural plant antioxidants, in the form of extracts and essential oils (EOs), have been obtained from different sources such as fruits (dragon fruit, guarana, and pomegranate), vegetables, (cabbage and onion), herbs and spices (epazot, ginger, rosemary, sage, thyme, turmeric and winter cider) by several extraction processes. However, in the context of current directives, there is a noticeable incentive for ‘green’ solvents to replace organic and conventional techniques to avoid harm to the environment, operators, and the health of consumers [44]. Nitrate and nitrite-rich natural extracts from sources such as red beet and celery can enhance the sensory properties of meat products while addressing safety and health concerns. The use of both natural sources of nitrate and nitrite to replace commercial nitrate and nitrite salts can develop the expected effects in terms of colour, lipid oxidation, microbial spoilage and safety, and sensory properties, but contrasting effects, in particular for redness and lipid oxidation, are still the main concerns. Advances in the utilization of these extracts support their progress towards higher-scale experiments and their extension towards the development of healthier and more functional meat products [53].

Consumers are becoming increasingly concerned about synthetic preservatives such as nitrites in meat, prompting the meat industry to explore alternatives to reduce nitrite levels. In these syntheses, the effects of hemp meal incorporation on the chemical and preservative characteristics of minced meat products with reduced nitrite content were investigated. Results of the study demonstrate that hemp flour can be utilized effectively as a natural ingredient with antioxidant properties in minced meat products, though some differences in sensory characteristics may occur [53]. Another study reported the effect of grape seed extract on colour stability, inhibition of lipid oxidation, and best overall acceptability after 6 days of storage in meat food products [54]. M. oleifera seed flour improved the physicochemical properties, organoleptic characteristics, consumer preference, and aerobic stability of cold storage of beef meat patties [55]. Onion (*Allium cepa* L.) is a widely cultivated and consumed vegetable due to its rich content of bioactive compounds. Red onion peel powder, which is a by-product derived from the onion industry, has attracted significant interest as a potential functional ingredient to improve the overall quality of food [56]. The bioactive compounds of pumpkin by-products, which are rich in phytochemicals, can prevent the oxidation process in different foods [57]. Avoidance of the use of certain additives may create situations where food safety is jeopardized. Some alternatives are based on the origin of the additive because naturalness is perceived as an essential characteristic for most consumers [3].

## 5. Influence of Natural and Artificial Additives on Food Perception

Modern consumers are tending towards products with natural additives and are increasingly focusing on the potential risks of consuming synthetic substances, reflecting current trends towards clean label foods. Certain demographic segments, however, are not influenced by these modern trends. Baby boomer consumers reject innovations in taste and tend towards the traditional, explained by a strong link with past food values. This highlights scepticism towards changes perceived as inauthentic [58]. While artificial flavours may be acceptable, artificial colours often evoke negative reactions, affecting the overall perception of the final product. Products with natural organoleptic properties are perceived as healthier, influencing consumer choices [59]. Consumers favour natural additives, their preference emphasizing the link between the type of additive and the quality of the product concerning the well-being of the human body [60]. Natural additives must meet strict food safety specifications similar to synthetic counterparts, complicating direct substitutions. Concerns about synthetic additives have led to increased scrutiny and demand for natural alternatives, although natural additives do not guarantee the safety of the final product [29,61].

Consumers prefer natural rather than artificial food additives, associating the latter with health risks. This perception influences purchasing decisions, as artificially named additives can lead to a perceived lack of naturalness, reducing acceptance of both the additive and the final product [62]. While consumers may prefer natural additives, studies show that artificial flavours can sometimes be more palatable [63]. Perceived quality differences between natural and artificial additives are shaped by cognitive integrations of the organoleptic properties of products, consumers associate natural additives with healthier products [64]. Educating consumers about the benefits of novel additives can stimulate positive emotional connections and acceptance of the final product [3]. Positive perceptions of the health benefits of additives can enhance emotional attachment [65]. The perceived health benefits of food additives can increase consumer acceptance by overcoming perceptions of risk. Consumers value convenience and time-saving aspects, stimulating emotional attachment to products, especially when additives are associated with positive attributes such as naturalness and safety, influencing purchasing decisions [66]. In contrast, scepticism about food additives persists, particularly among older demographics that favour traditional flavours. This scepticism may hinder acceptance, suggesting that consumer education and transparent labelling are key to promoting trust and acceptance of novel additives [58,61].

Consumers increasingly prefer natural additives to synthetic ones because of perceived health benefits. However, the presence of other additives in natural formulations may complicate this view, as consumers may not always recognize that natural does not inherently mean inherently safer or healthier. Direct replacement of synthetic colours requires technology development [67]. Consumers expressed concerns about food additives, with 63.7% linking these concerns to human health. This perception influences their purchasing decisions as they prefer products labelled as natural and free of artificial ingredients, indicating a high demand for clean label products [68]. Human health risk perceptions significantly influence their evaluation, attitude, and willingness to purchase, ultimately affecting their purchasing decisions on food products containing artificial additives [69].

## 6. Conclusions

Meat products are an essential component of the human diet, and the use of additives remains a widespread practice due to their impact on the overall quality of the final products. Some meat products require careful processing using various synthetic additives. The key aspect is to raise consumer awareness of the health impact of artificial additive and to encourage reformulation, more natural and healthier options for meat products. Consumers turn their attention to a clean label but are nonetheless guided by the sensory properties that the final product exhibits. Certain natural additives do not perform as well as synthetic additives in terms of the sensory properties of meat products. Synthetic additives are effective on the sensory properties of some meat products, but many studies confirm their harmful effects. The term artificial activates areas of the brain associated with rejection, even if the amount used under legislative standards states it is safe for consumption. The brain interprets multimodally involved information and provides an integrated, unified, and complex sensory experience about the product. The orbitofrontal cortex allows rapid associations between taste and other sensory stimuli. This helps to recognize foods based on previous experience, playing an important role in preference determination and food learning. Exploring the potential of natural additives to substitute artificial additives is an important step in accepting reformulated meat products.

Consumers associate additives with health effects, and the emotional response is the defining factor for product acceptability. Depending on demographic segments, consumers respond differently to products reformulated with new additives. How natural and artificial additives influence neurocognitive mechanisms and the perception of organoleptic properties of products explain consumption decisions. In general, consumers turn to the most natural products because they associate them with health benefits. The growing trend towards clean label products is committing the food industry to reformulate products to replace artificial additives with natural ingredients that have similar functions and productivity. Understanding food neurocognitive perception provides a complex, transparent framework. Optimizing the sensory experience for the development and market success of reformulated products helps to overcome consumer scepticism and contributes to the acceptance of additives, especially when they have significant results in the safety, quality, and nutritional value of the final food products that reach the consumer.

Future studies will focus on the impact on food quality, acceptability, and efficiency and will concentrate on overcoming the technical and economic hurdles in implementing natural alternatives. Challenges will include balancing the performance requirements of the food industry with consumer preferences for clean label products. This research will contribute to the efficient and sustainable development of safe and healthy meat products.

## Figures and Tables

**Table 1:** Natural replacements for most synthetic additives used in the meat industry.

Synthetic Additive	Natural Substitute	Effect on Meat Product	Ref.
**Sodium erythorbate**	Acerola Extract	Phenolic compounds; powerful antioxidant and an effective method in slowing down colour and lipid oxidation in meat products.	[39]
**Butylated hydroxyanisole (BHA)**	Rosemary Extract	Phenolic diterpenes, including carnosol, carnosic acid, epirosmanol, isorosmanol, rosmaridiphenol, and rosmariquinone contribute to the overall antioxidant activity of meat products and provide significant sensory properties.	[40,41,42]
**Butylated hydroxytoluene (BHT)**	Grape Seed Extract	Lipid stabilization of turkey meat; the addition of grape seed extract combined with vacuum packaging is considered a good method to improve lipid stability in cooked poultry meat.	[43]
**Sodium nitrite**	Beetroot and Celery Extracts	Contribute to the overall antibacterial activity of meat products.	[44]
**Monosodium** **glutamate**	Tomato by-products	Contain lycopene, which has been shown to have antioxidant activity; potentially improve sensory properties;	[45,46]
**Phosphoric acid**	Citrus lemon oil	Natural antioxidant and antimicrobial agent; beef has susceptibility to spoilage microorganisms during refrigeration, citrus lemon oil is an essential point for the use of natural antioxidants.	[47]
**Di- and triphosphates**	Papain; Ficin; Bromelain	Had significant results for meat tenderness and sensory properties.	[48,49]
**Polyphosphates**	Bamboo leaf Extract	The water-holding capacity and tenderness of poultry have been improved.	[50]

## Data Availability

No new data were created or analyzed in this study. Data sharing is not applicable to this article.
